# Altered Expression of Aromatase and Estrogen Receptors in Adipose Tissue From Men With Obesity or Type 2 Diabetes

**DOI:** 10.1210/clinem/dgaf038

**Published:** 2025-01-21

**Authors:** Fozia Ahmed, Susanne Hetty, Rutger Laterveer, Ece Busra Surucu, Argyri Mathioudaki, Edvin Hornbrinck, Vagia Patsoukaki, Johan Olausson, Magnus Sundbom, Maria K Svensson, Maria J Pereira, Jan W Eriksson

**Affiliations:** Department of Medical Sciences, Clinical Diabetology and Metabolism, Uppsala University, 751 85 Uppsala, Sweden; Department of Medical Sciences, Clinical Diabetology and Metabolism, Uppsala University, 751 85 Uppsala, Sweden; Department of Medical Sciences, Clinical Diabetology and Metabolism, Uppsala University, 751 85 Uppsala, Sweden; Department of Medical Sciences, Clinical Diabetology and Metabolism, Uppsala University, 751 85 Uppsala, Sweden; Department of Medical Sciences, Clinical Diabetology and Metabolism, Uppsala University, 751 85 Uppsala, Sweden; Department of Medical Sciences, Clinical Diabetology and Metabolism, Uppsala University, 751 85 Uppsala, Sweden; Department of Medical Sciences, Clinical Diabetology and Metabolism, Uppsala University, 751 85 Uppsala, Sweden; Department of Medical Sciences, Clinical Chemistry, Uppsala University, 751 85 Uppsala, Sweden; Department of Laboratory Medicine, Clinical Chemistry, Östersund Hospital, 831 31 Östersund, Sweden; Department of Surgical Sciences, Uppsala University, 751 85 Uppsala, Sweden; Department of Medical Sciences, Renal Medicine, Uppsala University, 751 85 Uppsala, Sweden; Department of Medical Sciences, Clinical Diabetology and Metabolism, Uppsala University, 751 85 Uppsala, Sweden; Department of Medical Sciences, Clinical Diabetology and Metabolism, Uppsala University, 751 85 Uppsala, Sweden

**Keywords:** aromatase, estrogen receptors, estradiol, adipose tissue, obesity, T2D

## Abstract

**Context:**

Obesity and insulin resistance in men are linked to decreased testosterone and increased estradiol (E2) levels. Aromatase (*ARO*) converts testosterone into E2, and this occurs mainly in adipose tissue in men. E2 acts through estrogen receptors *ESR1* and *ESR2*, and they potentially affect development of type 2 diabetes (T2D).

**Objective:**

This study explored alterations in *ARO*, *ESR1*, and *ESR2* in men with obesity or T2D.

**Methods:**

Subcutaneous adipose tissue (SAT) from men with or without obesity or T2D was analyzed for *ARO*, *ESR1*, and *ESR2* gene and protein expression. Data were compared across groups and correlated with markers of obesity, glycemia, insulin resistance, and sex hormones. Moreover, SAT was incubated with E2 or testosterone for ex vivo glucose uptake measurements.

**Results:**

*ARO* levels were higher in SAT from men with obesity compared to nonobese men, and gene expression correlated positively with adiposity, hyperglycemia, and insulin resistance. No association was found between *ARO* and circulating E2. Men with obesity had lower levels of *ESR1* and *ESR1:ESR2* ratio, but not *ESR2*. *ESR1* gene expression in SAT correlated negatively with adiposity and insulin resistance markers as well as with *ARO* expression, and tended to be lower in men with T2D. E2 reduced insulin-stimulated glucose uptake, while testosterone increased basal glucose uptake in adipocytes.

**Conclusion:**

Elevated *ARO* in SAT was found in obese men, and this was linked to insulin resistance and glycemia, supporting the idea that local estrogen production contributes to metabolic dysregulation. *ESR1* was reduced in men with T2D and was linked to adiposity and insulin resistance. Taken together, high *ARO* and altered *ESR1:ESR2* balance in SAT in obese men may contribute to insulin resistance and T2D development.

Obesity, which is a risk factor for the development of type 2 diabetes (T2D), is a major cause of morbidity and mortality globally (1). Although women are at a higher risk for obesity than men, men are more prone to obesity-related disorders such as insulin resistance and T2D (2, 3). This discrepancy is largely attributed to the higher tendency in men toward central adiposity (android fat distribution) compared to women (4). Android fat distribution is characterized by preferential fat storage both in subcutaneous and visceral adipose tissue (SAT and VAT, respectively) in the abdominal region, and is associated with increased cardiometabolic risk, independent of body mass index (BMI). In contrast, lower-body obesity (gynoid fat distribution), more common in premenopausal women, is considered metabolically favorable, carrying a lower risk for glucose and lipid dysregulation (2, 4).

Sex differences in body fat distribution have been associated with differences in estrogen levels (5). While estrogen is crucial for regulating fat distribution in women, it also plays a pivotal role in regulating fat distribution in men (6, 7). In women, the main estrogen, estradiol (E2), promotes a gynoid fat distribution, but, in contrast, hyperestrogenemia in men is associated with increased visceral fat mass and obesity (7), highlighting sex-specific effects of estrogen on body fat distribution. In men, approximately 15% of circulating estrogens are derived from testicular production, whereas the remainder is produced in peripheral tissues through conversion of androgens by the enzyme aromatase (ARO) (8), encoded by the *CYP19A1* (or *ARO*) gene. Adipose tissue is a major source of estrogen production in men, and it is hypothesized that increased aromatase activity in adipose tissue contributes to lower testosterone and hyperestrogenemia in men with obesity (8, 9).

Estrogens exert their effects mainly by binding to their canonical nuclear receptors, estrogen receptor α (ESR1) and β (ESR2). In adipose tissue, ESR1 plays an important role both in adipocyte glucose and lipid metabolism by promoting adipocyte glucose uptake and stimulating lipolysis (10-12). Moreover, obese hypogonadal men with hyperestrogenemia have reduced adipose tissue expression of ESR1 (7). The role of ESR2 on adipocyte glucose and lipid metabolism is more complex, with some studies suggesting a role in insulin resistance (13, 14), whereas others demonstrate a protective role against adiposity and insulin resistance (15). In a previous study, we have shown that E2 directly inhibits glucose uptake in SAT from late postmenopausal women, whose metabolic profiles are more similar to men than premenopausal women, and this effect was at least partially mediated by ESR2 (14). Whether E2 has a similar effect in men, and whether the local ARO-estrogen-ESR axis contributes to insulin resistance in SAT in obesity in men, remains to be fully elucidated.

In this study, we aimed to investigate the role of *ARO*, *ESR1*, and *ESR2* in SAT from men with obesity and T2D. Moreover, we investigate the effects of E2 and testosterone on glucose uptake in adipocytes isolated from SAT from men.

## Materials and Methods

### Participants

Cohort 1 included 77 men (BMI 21.3-43.8 kg/m^2^, aged 18-77 years) without T2D that were subcategorized according to obesity status (lean: BMI <25; overweight: BMI 25.0-29.9; obesity: BMI ≥30). Cohort 2 included 17 men with T2D (BMI 25.0-39.1 kg/m^2^, aged 45-71 years). From cohorts 1 and 2, SAT biopsies were obtained by needle aspiration from the lower part of the abdomen after local dermal anesthesia with lidocaine (Xylocaine, AstraZeneca). In addition, paired SAT and VAT surgical biopsies were obtained from 13 men (cohort 3) without T2D undergoing kidney donation at Uppsala University Hospital. These individuals were used only for comparisons of SAT and VAT differences in *ARO*, *ESR1*, and *ESR2* gene expression.

Individuals with type 1 diabetes, other endocrine disorders, cancer, or other major illnesses, as well as ongoing medication with β-adrenergic blockers, systemic glucocorticoids, or immune-modulating therapies, were excluded from the study. All participants with T2D were on a stable dose of metformin as their only antidiabetic medication. Anthropometrics were measured and blood samples were taken after an overnight fast (>10 hours) for measurements of glycated hemoglobin A_1c_ (HbA_1c_), glucose, lipids, and insulin levels. Total body fat percentage was assessed with bioimpedance (Body Composition Analyzer BC-418; Tanita). In a subset of participants (n = 19), an oral glucose tolerance test was conducted to analyze plasma glucose, insulin, glycerol, and free fatty acids. Serum sex hormone levels were measured in a subgroup of individuals from cohort 1 (n = 56) and cohort 2 (n = 10) on the Cobas pro e801 instrument (Roche Diagnostics) at the Department of Clinical Chemistry, Östersund Hospital. Free androgen index (FAI) was calculated as (testosterone × 100)/(sex hormone–binding globulin [SHBG]) as previously described (16), free estradiol (E2) index was calculated as (E2 × 100)/(272.11 × SHBG) as previously described (17), and the total testosterone/total E2 ratio was calculated (both in nmol/L). The clinical and anthropometric characterization of participants in cohorts 1 and 2 are presented in Table 1, and cohort 3 in Supplementary Table S1 (18).

**Table 1.** Anthropometric and clinical characteristics of participating men

Variable	No diabetes, cohort 1			T2D, cohort 2
	Lean	Overweight	Obese	
No.	14	39	24	17
Age, y	43 ± 18	48 ± 18	51 ± 15	61 ± 6
BMI, kg/m^2^	23.4 ± 1.2	27.1 ± 1.3	33.5 ± 3.3	30.3 ± 4.1
WHR	0.91 ± 0.07	0.94 ± 0.08	1.0 ± 0.06	1.03 ± 0.04
Body fat, %	18.5 ± 5.2	23.6 ± 5.0	30.2 ± 4.5	29.3 ± 5.4
P-glucose, mmol/L	5.6 ± 0.5	5.7 ± 0.5	5.9 ± 0.7	8.3 ± 1.3
HbA_1c_, mmol/mol	33.8 ± 3.7	34.4 ± 3.7	35.2 ± 3.4	50.1 ± 7.1
S-insulin, mU/L	7.5 ± 6.9	9.4 ± 65.8	12.6 ± 5.6	17.5 ± 9.4
HOMA-IR	1.9 ± 1.8	2.3 ± 1.4	3.3 ± 1.8	6.3 ± 3.2
P-total cholesterol, mmol/L	5.2 ± 1.1	5.0 ± 1.1	5.2 ± 0.9	4.5 ± 0.9
P-HDL cholesterol, mmol/L	1.4 ± 0.3	1.3 ± 0.3	1.2 ± 0.3	1.1 ± 0.2
P-LDL cholesterol, mmol/L	3.4 ± 0.9	3.2 ± 1.0	3.5 ± 0.6	2.8 ± 0.8
P-triglycerides, mmol/L	1.1 ± 0.6	1.3 ± 0.7	1.5 ± 0.8	1.5 ± 0.6
S-DHEAS, µmol/L*^a^*	7.1 ± 4.1	6.9 ± 2.8	6.0 ± 3.5	4.5 ± 2.0
S-SHBG, nmol/L*^a^*	39.4 ± 8.8	35.8 ± 12.6	32.5 ± 12.7	30.3 ± 19.3
S-Testosterone, nmol/L*^a^*	18.3 ± 4.2	18.5 ± 6.1	15.7 ± 8.1	12.8 ± 2.6
S-E2, pmol/L)*^a^*	99.7 ± 29.2	121 ± 40.8	131 ± 71.4	107 ± 37.0
Free androgen index*^a^*	48 ± 18	55 ± 22	56 ± 42	42 ± 10
Free estradiol index*^a^*	1.0 ± 0.5	1.4 ± 0.7	1.8 ± 1.4	1.4 ± 0.5
Testosterone/E2 ratio*^a^*	193 ± 59	161 ± 57	123 ± 27	121 ± 33
FSH, IU/L*^a^*	4.6 ± 1.8	6.3 ± 6.5	5.3 ± 3.6	8.3 ± 6.5
LH, IU/L*^a^*	5.2 ± 1.8	6.4 ± 2.9	5.0 ± 2.0	7.1 ± 3.6

Data are mean ± SD. Blood chemistry is fasting.

Abbreviations: BMI, body mass index; DHEAS, dehydroepiandrosterone sulfate; E2, estradiol; FAI, free androgen index; FEI, free estrogen index; FSH, follicle-stimulating hormone; HbA_1c_, glycated hemoglobin A_1c_; HDL, high-density lipoprotein; HOMA-IR, homeostatic model assessment of insulin resistance; LDL, low-density lipoprotein; LH, luteinizing hormone; P, plasma; S, serum; SHGB, sex hormone–binding globulin; T2D, type 2 diabetes; WHR, waist-hip ratio.

^*a*^Cohort 1 n = 11/27/18 (lean/overweight/obese); cohort 2 n = 10.

One part of adipose tissue biopsies was snap-frozen in liquid nitrogen and used for quantitative polymerase chain reaction (qPCR) and Western blot analysis. The other part was used to perform ex vivo glucose uptake in freshly isolated adipocytes or following 24 hours’ incubation with E2 and testosterone, to evaluate their effects on glucose uptake. Not all experiments could be performed in every participant due to the limited amount of adipose tissue. In total, samples from 69 individuals (cohorts 1-3) were used for gene expression analyses, 34 for protein analyses using Western blot (cohorts 1 and 2), and 18 and 16 for the glucose uptake experiments in freshly isolated adipocytes (cohorts 1 and 2) or after 24-hour incubation with E2 and testosterone (cohort 1), respectively.

All participants were recruited at the Uppsala University Hospital. The regional ethics review board in Uppsala (Dnr 2013/330, Dnr 2013-183/494, Dnr 2014/313, Dnr 2014/255, Dnr 2018/385, Dnr 2020-06350) approved the studies, and all participants gave their written informed consent.

### Real-Time Quantitative Polymerase Chain Reaction

Total RNA was extracted from whole adipose tissue (n = 69) using the RNeasy lipid tissue mini kit (Qiagen) as previously described (14). RNA (400 ng) was reverse-transcribed using a High-Capacity cDNA Reverse Transcription Kit (Applied Biosystems, Thermo Fisher Scientific). Protocols were carried out as per manufacturer's guidelines. qPCR gene expression analysis was performed using TaqMan gene expression assays (Thermo Fisher; gene names and Taqman probes are listed in Supplementary Table S2 (18)) on the QuantStudio 3 sequence detection system (Applied Biosystem) and calculated using the 2^−ΔCT^ method. Gene expression levels were normalized to the housekeeping gene glucuronidase β, *GUSB*. All samples were run in duplicates.

### Western Blot

Total protein was isolated from SAT of 34 individuals with and without obesity or T2D (from cohorts 1 and 2), covering a wide range of BMI and insulin sensitivity, and was used for Western blot analysis of ARO, ESR1, and ESR2 protein levels. A subgroup of these participants without T2D was used for comparing lean men and men with obesity (matched for age). Another subgroup of participants was used to compare men with or without T2D (matched for age and BMI).

Proteins were separated by sodium dodecyl sulfate–polyacrylamide gel electrophoresis as previously reported (19), and transferred to poly(vinylidene fluoride) membranes. Membranes were incubated overnight with the primary antibodies antiaromatase (Abcam catalog No. ab35604, RRID:AB_867729; 1:500), anti-ESR1 (Cell Signaling Technology catalog No. 13258, RRID:AB_2632959; 1:1000), or anti-ESR2 (Thermo Fisher Scientific catalog No. MA5-24807, RRID:AB_2717280; 1:500) and protein levels were quantified as described in Supplementary methods (18) and normalized to total protein using stain-free technology (Bio-Rad) (stain-free total protein blots are shown in Supplementary Fig. S1 (18)).

### Immunohistochemistry

Immunofluorescence analysis of ARO in SAT from men were performed on formalin-fixed, paraffin-embedded SAT sections (5 μm) according to standard protocols. SAT sections were deparaffinized and rehydrated, followed by heat-induced antigen retrieval in 10 mM sodium citrate buffer (pH 6.0). Slides were washed 2× for 5 minutes with phosphate-buffered saline (PBS) and 0.05% Triton-X-100 (PBS-T) and then blocked in PBS-T with 5% normal goat serum for 1 hour at room temperature. Sections were incubated with the primary antibody antiaromatase (Abcam, RRID:AB_867729, 1:100) overnight in a humidified chamber, followed by secondary antibody incubation (Thermo Fisher Scientific catalog No. A-11008, RRID:AB_143165), 1:250) for 2 hours at room temperature. ProLong Diamond Antifade Mountant with DAPI (4′,6-diamidino-2-phenylindole), for counterstaining of cell nuclei, was used for mounting the slides (Invitrogen). Sections were imaged using brightfield imaging as well as DAPI (for nuclear stain detection) and fluorescein isothiocyanate (for aromatase detection) filter cubes with digital confocal, for fluorescence imaging, with 20× magnification using the ImageXpress Pico Automated Cell Imaging System (Molecular Devices).

### Adipose Tissue Incubations and Glucose Uptake in Adipocytes

Glucose uptake was measured in 2 conditions: 1) in freshly isolated adipocytes from participants with and without T2D (cohorts 1 and 2, n = 18, BMI: 22.8-39.1, aged 34-71 years) for correlations with *ARO*, *ESR1*, and *ESR2* gene expression, and 2) in adipocytes isolated from SAT from participants without T2D after 24-hour incubation with or without E2 or testosterone (cohort 1, n = 16, BMI 23.7-43.8, aged 23-77 years; clinical characteristics of participants are shown in Supplementary Table S3 (18)).

For the incubations, SAT was incubated at 37 °C for 24 hours in phenol red-free Dulbecco's Modified Eagle Medium (DMEM) media containing 6 mM glucose (Invitrogen Corporation), 10% charcoal-stripped fetal bovine serum (FBS, Invitrogen), and 1% penicillin-streptomycin (PEST, Invitrogen). The medium was supplemented with either 0.1 nM (physiological) E2 (Sigma) or 100 nM of testosterone (Sigma), or left untreated (control). After incubation, adipocytes were isolated from the incubated tissue for glucose uptake measurements.

Glucose uptake was measured using radiolabeled glucose as previously described (14, 20) and detailed in Supplementary methods (18). Glucose uptake was performed under basal conditions or with insulin stimulation (25 and 1000 µU/mL) and was expressed as clearance of medium glucose per cell. Cell number was determined following measurements of triglyceride content (Doles extraction) (21), and cell size (Axiovision) (22).

### Single-Nucleotide Variation Analysis

Associations of *CYP19A1* single-nucleotide variations (SNVs; formerly single-nucleotide polymorphisms) with diabetes and obesity-related traits were assessed in men. Summary statistics were downloaded from the NHGRI-EBI GWAS Catalog (23) March 20, 2024, for the gene *CYP19A1* and after trait selection, additional data were downloaded for study GCST012228 (24).

### Statistics

All data are presented as mean ± SEM, unless otherwise stated. The normality of data was first assessed using the Shapiro-Wilk test. Nonnormally distributed data were log-transformed prior to statistical testing. Two-sample *t* tests were used for analyses between 2 independent groups, and paired *t* tests were applied for paired group analysis. One-way analysis of variance was used for comparisons between more than 2 groups. The false discovery rate method Benjamini, Krieger, and Yekutieli was used for multiple comparison correction. The Spearman correlation was used to test for bivariate analysis, and relevant statistically significant variables were included in multilinear regression models to predict the effect of clinical variables on *ARO*, *ESR1*, and *ESR2* gene expression levels. A *P* value less than .05 was considered statistically significant. All data were analyzed using GraphPad Prism 10.3.1, IBM SPSS version 28.0.1.0.

## Results

### Expression of *ARO*, *ESR1*, and *ESR2* in Subcutaneous Adipose Tissue From Men With Obesity and Type 2 Diabetes

**Figure 1.**
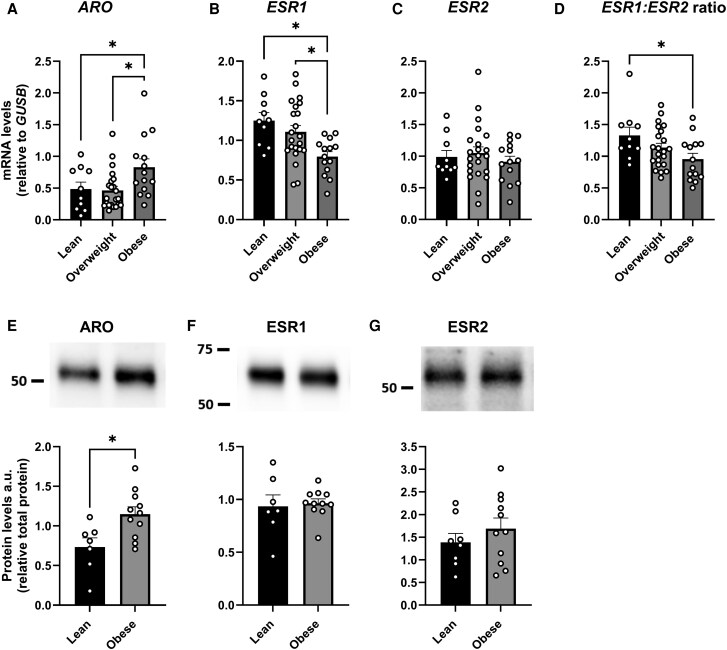
Gene and protein expression of ARO, ESR1, and ESR2 in subcutaneous adipose tissue in men with or without obesity. Gene and protein expression of A and E, ARO; B and F, ESR1; C and G, ESR2; and D, *ESR1:ESR2* gene expression ratio in lean men or men with overweight or obesity from cohort 1 (gene expression: n = 10/22/14, lean/overweight/obese; protein expression: n = 7/11, lean/obese; groups matched for age) *P less than .05. Data are mean ± SEM.

Gene and protein expression of *ARO*, *ESR1*, and *ESR2* in SAT from participants with or without obesity (matched for age) or T2D (matched for age and BMI) was first assessed. *ARO* gene expression was higher in SAT from men with obesity compared both to lean men and men with overweight (Fig. 1A; *P* < .05). Protein levels were also higher in men with obesity compared to lean men (Fig. 1E; *P* < .05). *ESR1* gene expression levels were lower in men with obesity than in lean men and men with overweight (Fig. 1B; *P* < .05), while *ESR2* gene expression levels were not affected by obesity status (Fig. 1C). *ESR1:ESR2* gene expression ratio was lower in participants with obesity compared to lean individuals (Fig. 1D; *P* < .05). Neither ESR1 nor ESR2 protein levels were affected by obesity status (Fig. 1F and 1G).

**Figure 2.**
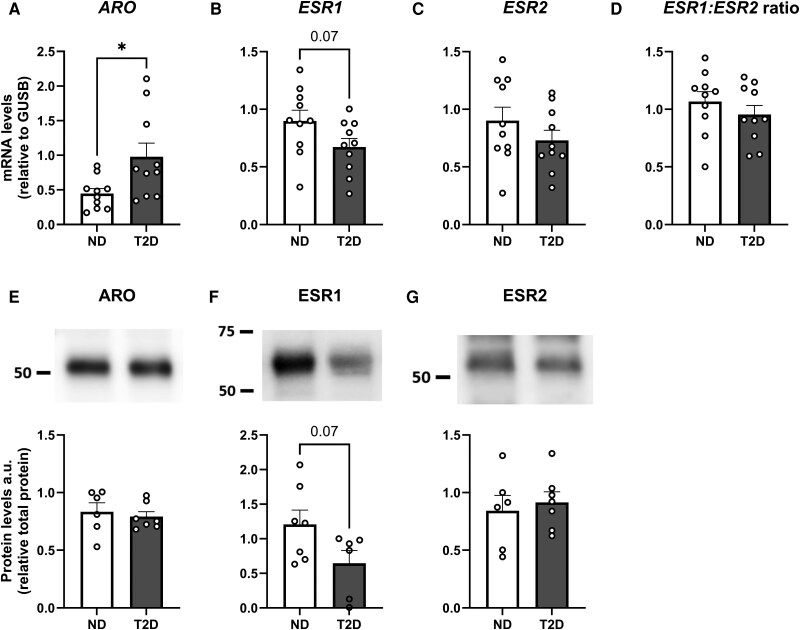
Gene and protein expression of ARO, ESR1, and ESR2 in subcutaneous adipose tissue in men with or without type 2 diabetes (T2D). Gene and protein expression of A and E, ARO; B and F (F is composite of two separate parts of the blot), ESR1; C and G, ESR2; and D, *ESR1:ESR2* gene expression ratio in individuals with no diabetes (ND) or with T2D from cohorts 1 and 2, respectively (gene expression: n = 10/10, protein expression: n = 6/7; ND/T2D; groups matched for age and body mass index). *P less than .05. Data are mean ± SEM.

The *ARO* gene, but not protein expression levels, were higher in participants with T2D compared to individuals with no diabetes (ND) (Fig. 2A; *P* < .05 and Fig. 2E; *P* > .05). *ESR1* gene and protein expression in individuals with T2D showed a nominal trend toward being lower in participants with T2D compared to ND individuals (Fig. 2B and 2F; both *P* = .07). Neither *ESR2* gene nor protein expression levels were affected by T2D (Fig. 2C and 2G; *P* > .05). *ESR1:ESR2* gene expression ratio was not different between ND and subjects with T2D (Fig. 2D).

### Association Between the Gene Expression Levels of *ARO*, *ESR1*, and *ESR2* in Subcutaneous Adipose Tissue

**Figure 3.**
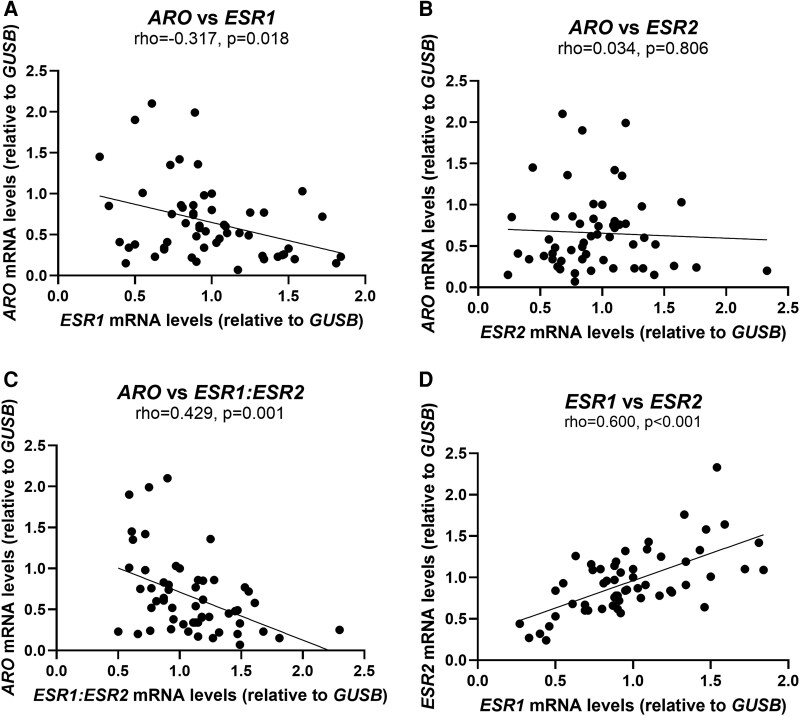
Association between the gene expression levels of *ARO*, *ESR1*, and *ESR2* in subcutaneous adipose tissue (SAT). Association between gene expression of A, *ARO* and *ESR1*; B, *ESR2*; and C, *ESR1:ESR2* gene expression. D, Association between gene expression of *ESR1* and *ESR2* in SAT. Cohorts 1 and 2 including men with and without type 2 diabetes, n = 56. Correlations are Spearman rho.

We assessed whether there was an association between the gene expression of *ARO*, *ESR1*, and *ESR2* in the combined cohorts 1 and 2, which included men with or without obesity or T2D. *ARO* gene expression was negatively associated with *ESR1* and *ESR1:ESR2* expression (Fig. 3A and 3C; *P* < .05) but not *ESR2* expression (Fig. 3B)*. ESR1* gene expression was positively associated with *ESR2* gene expression (Fig. 3D; *P* < .001). These associations were similar when participants were subdivided by obesity status and T2D status (data not shown).

### Association Between *ARO*, *ESR1*, *ESR2*, and *ESR1:ESR2* Ratio With Age, Markers of Obesity, and Insulin Resistance, and Sex Hormones

**Figure 4.**
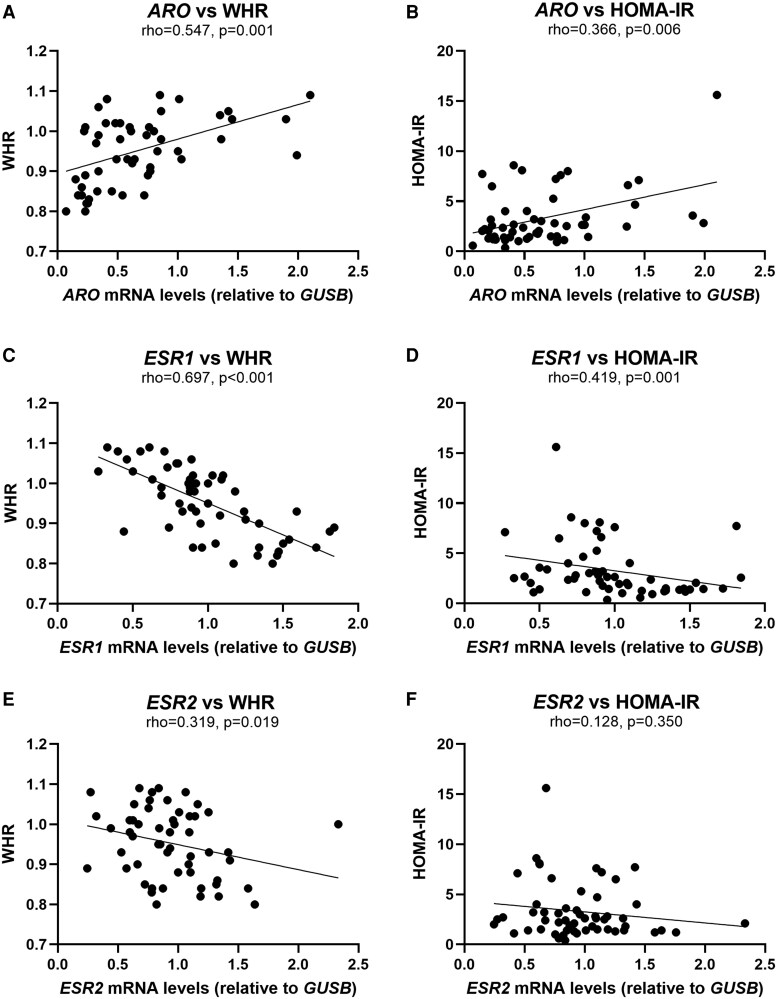
Association between the gene expression levels of *ARO*, *ESR1*, and *ESR2* in subcutaneous adipose tissue with waist-hip ratio (WHR) and homeostatic model assessment of insulin resistance (HOMA-IR). Association between WHR and gene expression of A, *ARO*; C, *ESR1*; E, *ESR2*, and between HOMA-IR and gene expression of B, *ARO* and D, *ESR1*; F, *ESR2*. Cohorts 1 and 2, including men with and without type 2 diabetes, n = 56. Correlations are Spearman rho.

Correlations between *ARO*, *ESR1*, *ESR2*, and *ESR1:ESR2* gene expression in SAT with markers of obesity and insulin resistance, sex hormones, and ex vivo glucose uptake (Table 2 and Fig. 4) were performed in the combined cohorts 1 and 2, including individuals with or without obesity and T2D, to ensure comprehensive coverage across a wide range of obesity and insulin resistance. *ARO* gene expression correlated positively with age, markers of obesity (eg, BMI, waist-hip ratio [WHR; see Fig. 4A], body fat percentage), and markers of hyperglycemia and insulin resistance (eg, HbA_1c_, homeostatic model assessment of insulin resistance [HOMA-IR] (Fig. 4B), fasting insulin) (*P* < .05). Moreover, *ARO* gene expression was negatively associated with the Matsuda index, a marker of insulin sensitivity (*P* < .01). In individuals without T2D, ARO protein levels also correlated positively with the obesity markers BMI (rho = .554; *P* = .014) and body fat percentage (rho = 0.625; *P* = .013), as well as markers of insulin resistance such as fasting insulin (rho = 0.505; *P* = .033) and a positive trend with HOMA-IR (rho = 0.473; *P* = .073) (data not shown).

**Table 2.** Correlations between *ARO*, *ESR1*, *ESR2*, and *ESR1:ESR2* gene expression in subcutaneous adipose tissue and clinical characteristics, and expression of adipose tissue metabolic genes

	*ARO*		*ESR1*		*ESR2*		*ESR1:ESR2*	
	Rho	*P*	Rho	*P*	Rho	*P*	Rho	*P*
Age	**0**.**404**	.**002**	**−0**.**459**	**<**.**001**	−0.067	.621	**−0**.**371**	.**005**
Adiposity measures								
BMI	**0**.**424**	.**001**	**−0**.**466**	**<**.**001**	−0.118	.388	**−0**.**293**	.**028**
WHR	**0**.**541**	**<**.**001**	**−0**.**697**	**<**.**001**	**−0**.**319**	.**019**	**−0**.**348**	.**010**
Body fat percentage*^a^*	**0**.**689**	**<**.**001**	**−0**.**646**	**<**.**001**	−0.231	.163	**−0**.**350**	.**031**
Adipocyte cell size*^b^*	−0.290	.229	−0.093	.705	−0.164	.502	0.136	.579
Hyperglycemia and insulin resistance								
HbA_1c_	**0**.**323**	.**016**	**−0**.**352**	.**008**	−0.129	.348	−0.235	.083
HOMA-IR	**0**.**366**	.**006**	**−0**.**419**	.**001**	−0.128	.350	−0.226	.097
Matsuda index*^c^*	**−0**.**614**	.**002**	0.084	.710	−0.118	.601	0.343	.118
Fasting glucose	0.158	.248	−0.183	.182	0.101	.462	**−0**.**282**	.**037**
Fasting insulin	**0**.**385**	.**003**	**−0**.**434**	.**001**	−0.159	.243	−0.207	.126
Sex hormones								
Testosterone	−0.169	.212	**0**.**466**	**<**.**001**	**0**.**297**	.**026**	0.107	.432
E2	−0.008	.956	0.023	.828	0.026	.851	−0.010	.942
Testosterone/E2 ratio	−0.193	.157	**0**.**371**	.**005**	*0*.*248*	.*066*	0.069	.641
FAI	*−0*.*231*	.*087*	**0**.**297**	.**026**	0.066	.630	*0*.*246*	.*068*
FEI	−0.078	.566	−0.093	.494	−0.144	.289	0.103	.448
DHEAS	−0.187	.168	*0*.*226*	.*094*	−0.061	.656	*0*.*232*	.*085*
SHBG	0.043	.752	0.173	.202	0.222	.100	−0.114	.401
Ex vivo glucose uptake								
Basal*^d^*	−0.380	.110	**0**.**606**	.**008**	*0*.*458*	.*058*	0.089	.756
Insulin 25 µU/mL*^d^*	−0.321	.194	**0**.**598**	.**009**	**0**.**506**	.**032**	0.005	.989
Insulin 1000 µU/mL*^d^*	−0.357	.145	**0**.**585**	.**011**	**0**.**471**	.**048**	0.036	.887
Adipose tissue metabolic genes								
Glucose transporter 4, *GLUT4*	*−0*.*234*	.*088*	**0**.**750**	**<**.**001**	**0**.**527**	**<**.**001**	0.145	.292
Insulin receptor substrate 1, *IRS1*	**−0**.**265**	.**049**	**0**.**726**	**<**.**001**	**0**.**598**	**<**.**001**	0.104	.445
Hormone sensitive lipase, *HSL*	**−0**.**305**	.**026**	**0**.**555**	**<**.**001**	**0**.**571**	**<**.**001**	−0.026	.856
Adipose triglyceride lipase, *ATGL*	−0.035	.808	**0**.**639**	**<**.**001**	**0**.**354**	.**009**	0.225	.102
Fatty acid synthase, *FASN*	−0.175	.210	**0**.**428**	**<**.**001**	**0**.**426**	.**002**	−0.021	.887
Diacylglycerol O-acyltransferase 2, *DGAT2*	0.128	.386	**0**.**666**	.**002**	**0**.**448**	**<**.**001**	0.179	.196

N = 56, unless otherwise stated. Table presents Spearman rho correlation coefficient. Statistically significant correlation values are shown in bold, *P* less than .05; and italics, *P* less than .1.

SAT obtained from individuals with and without obesity and T2D cohorts 1 and 2.

Abbreviations: BMI, body mass index; DHEAS, dehydroepiandrosterone sulfate; E2, estradiol; FAI, free androgen index; FEI, free estrogen index; HbA_1c_, glycated hemoglobin A_1c_; HOMA-IR: homeostatic model assessment of insulin resistance; HDL, high-density lipoprotein; LDL, low-density lipoprotein; SAT, subcutaneous adipose tissue; SHGB, sex hormone–binding globulin; WHR, waist-hip ratio; T2D, type 2 diabetes.

^*a*^n = 38.

^*b*^n = 33.

^*c*^n = 22.

^*d*^n = 18.

*ESR1* gene expression inversely correlated with age, obesity markers (eg, BMI, WHR [Fig. 4C], body fat percentage), and markers of hyperglycemia and insulin resistance (eg, HbA_1c_, HOMA-IR [Fig. 4D], insulin) (*P* < .05) (see Table 2). *ESR2* gene expression in SAT correlated negatively with WHR (Fig. 4E), while no correlations were found with markers of hyperglycemia and insulin resistance (eg, HOMA-IR [Fig. 4F]). *ESR1:ESR2* gene expression was negatively associated with age, obesity markers (eg, BMI, WHR, body fat percentage), and fasting glucose. Circulating testosterone was positively associated with *ESR1* and *ESR2* gene expression in SAT (*P* < .05).

We also assessed whether *ARO*, *ESR1*, *ESR2*, and *ESR1:ESR2* gene expression were associated with ex vivo adipocyte glucose uptake (see Table 2). *ESR1* gene expression was positively associated with basal and insulin-stimulated adipocyte glucose uptake (*P* < .05), while *ESR2* was positively associated with insulin-stimulated adipocyte glucose uptake (*P* < .05). *ARO* gene expression was not associated with basal or insulin-stimulated glucose uptake.

Moreover, we performed correlation analysis for gene expression of *ARO*, *ESR1*, *ESR2*, and *ESR1:ESR2* with expression of key genes for adipocyte metabolism in SAT (see Table 2). Strong positive correlations were found between both *ESR1* and *ESR2* with genes involved in glucose uptake (*GLUT4*, *IRS1* [all *P* < .001], lipolysis [*HSL*, both *P* < .001]; *ATGL*; *P* < .001 and *P* = .009, respectively) and de novo lipogenesis (*FASN, P* < .001 and *P* = .002, respectively; *DGAT2*; *P* = .002 and *P* < .001). *ARO* correlated negatively with *IRS1* (*P* < .049), and there was also a negative trend with *GLUT4* (*P* = .088). Moreover, *ARO* was negatively correlated with *HSL* (*P* = .026). No statistically significant correlations were found between *ESR1:ESR2* ratio and any of the adipocyte metabolism genes measured.

### Multilinear Regression Modeling of Factors Associated With *ARO*, *ESR1*, and *ESR2* Expression in Subcutaneous Adipose Tissue

To determine which clinical characteristics were the strongest predictors of *ARO*, *ESR1*, and *ESR2* expression in SAT, we performed multilinear regression models including variables with statistically significant correlations. Model A consisted of age, BMI, WHR, HbA_1c_, and HOMA-IR, and model B included age, HbA_1c_, HOMA-IR, and body fat percentage (Table 3). Body fat percentage could not be included in a multilinear regression model with either BMI or WHR due to collinearity. In model A, WHR was the strongest predictor of *ARO* (std β = .568; *P* = .013), *ESR1* (std β = −.650; *P* < .001), and *ESR2* (std β = −.605; *P* = .012). Variables included in model A were not statistically significant predictors of *ESR1:ESR2* gene expression. In model B, body fat percentage was the strongest predictor of *ARO* (std β = .528; *P* = .014) and *ESR1* (std β = −.577; *P* = .004). Variables included in model B were not statistically significant predictors of *ESR2* gene expression or the *ESR1:ESR2* ratio. Also, when substituting HbA_1c_ with T2D status in models A and B, WHR and body fat percentage, respectively, remained the only statistically significant predictors of both *ARO* and *ESR1* (data not shown).

**Table 3.** Multilinear regression models for predicting *ARO*, *ESR1*, *ESR2*, and *ESR1:ESR2* gene expression in subcutaneous adipose tissue

	*ARO*		*ESR1*		*ESR2*		*ESR1:ESR2*	
Model A	*R^2^: 0.247*		*R^2^: 0.496*		*R^2^: 0.171*		*R^2^: 0.242*	
	Standard β	*P*	Standard β	*P*	Standard β	*P*	Standard β	*P*
Age	−0.357	.086	−0.009	.957	0.276	.202	−0.306	.140
BMI	0.156	.384	−0.078	.596	0.095	.614	−0.162	.368
WHR	**0**.**568**	.**013**	**−0**.**650**	**<**.**001**	**−0**.**605**	.**012**	−0.073	.744
HbA_1c_	0.033	.865	−0.162	.306	−0.095	.638	−0.141	.469
HOMA-IR	−0.032	.865	0.178	.254	0.046	0.817	0.127	.504

Model B	*R^2^: 0.287*		*R^2^: 0.389*		*R^2^: 0.074*		*R^2^: 0.257*	
	Standard β	*P*	Standard β	*P*	Standard β	*P*	Standard β	*P*
Age	−0.053	.795	−0.144	.449	0.170	.468	−0.420	.052
HbA_1c_	−0.06	.781	0.071	.723	−0.034	.890	0.076	.731
HOMA-IR	0.127	.537	0.049	.795	−0.043	.852	0.051	.809
Body fat percentage*^a^*	**0**.**528**	.**014**	**−0**.**577**	.**004**	−0.304	.197	−0.192	.360

N = 56, unless otherwise stated. Subcutaneous adipose tissue obtained from individuals with and without obesity and T2D cohorts 1 and 2. Bold indicates statistical significance.

Abbreviations: BMI, body mass index; HbA_1c_, glycated hemoglobin A_1c_; HOMA-IR, homeostatic model assessment of insulin resistance; T2D, type 2 diabetes; WHR, waist-hip ratio.

^*a*^n = 38.

To evaluate if *ARO*, *ESR1*, *ESR2*, or *ESR1:ESR2* ratio was a predictor of metabolic parameters, we performed multilinear regression models with HOMA-IR or WHR as dependent variables. The strongest predictor of WHR was *ARO* (R^2^ = 0.563, β = .221; *P* = .033), while *ESR1*, *ESR2*, and *ESR1:ESR2* ratio were not statistically significant predictors. HOMA-IR with the same predictors did not yield a significant model.

### Association Between Sex Hormone Levels in Blood and Markers of Obesity and Insulin Resistance

We assessed the association between sex hormones in blood with age, markers of obesity, and insulin resistance (Table 4). Testosterone correlated negatively with age, obesity markers (eg, BMI, WHR, body fat percentage), and markers of insulin resistance and hyperglycemia (eg, HbA_1c_, HOMA-IR, serum insulin). E2 was not statistically significantly associated with these clinical markers of obesity and insulin resistance. DHEAS was negatively associated with age, markers of obesity (eg, WHR, body fat percentage), and HbA_1c_ (*P* < .05). SHBG was negatively associated with insulin (*P* < .05). Since the majority of testosterone and E2 are not found as free molecules, we calculated the free androgen and estrogen index, FAI and FEI, respectively. FAI correlated negatively with markers of obesity (eg, WHR, body fat percentage), insulin resistance, and impaired glycemia (eg, HbA_1c_, HOMA-IR, insulin), and positively with cell size and basal and insulin-stimulated adipocyte glucose uptake (*P* < .05).

**Table 4.** Association between sex hormones in blood and age, markers of obesity, impaired glycemia, and insulin resistance

	Testosterone		E2		DHEAS		SHBG		FAI		FEI	
	Rho	*P*	Rho	*P*	Rho	*P*	Rho	*P*	Rho	*P*	Rho	*P*
Age	**−0**.**336**	.**005**	0.010	.939	**−0**.**695**	**<**.**001**	0.240	.051	**−0**.**612**	**<**.**001**	−0.198	.109
Adiposity measures												
BMI	**−0**.**308**	.**011**	0.107	.388	*−0*.*229*	.*062*	−0.196	.111	−0.098	.429	*0*.*226*	.*066*
WHR	**−0**.**494**	**<**.**001**	−0.027	.829	**−0**.**480**	**<**.**001**	0.047	.708	**−0**.**517**	**<**.**001**	−0.066	.602
Body fat percentage*^a^*	**−0**.**404**	.**004**	−0.032	.828	**−0**.**467**	.**001**	−0.076	.603	**−0**.**365**	.**010**	0.026	.858
Cell size*^b^*	−0.119	.627	−0.318	.185	0.178	.467	−0.403	.087	**0**.**508**	.**026**	0.172	.481
Hyperglycemia and insulin resistance												
HbA_1c_	**−0**.**377**	.**002**	−0.035	.782	**−0**.**441**	**<**.**001**	0.071	.571	**−0**.**444**	**<**.**001**	−0.102	.417
HOMA-IR	**−0**.**547**	**<**.**001**	−0.070	.579	−0.165	.188	*−0*.*239*	.*056*	**−0**.**292**	.**018**	0.104	.408
Matsuda index*^c^*	−0.166	.460	*0*.*402*	.*064*	−0.071	.755	−0.147	.513	−0.039	.863	0.377	.084
Fasting glucose	*−0*.*243*	.*051*	0.001	.991	*−0*.*206*	.*099*	0.046	.715	*−0*.*232*	.*062*	0.012	.923
Fasting insulin	**−0**.**540**	**<0**.**001**	−0.060	.630	−0.114	.364	**−0**.**271**	.**028**	**−0**.**265**	.**032**	0.120	.336
Glucose uptake												
Basal*^d^*	0.278	.265	0.162	.521	−0.032	.900	−0.040	.874	**0**.**501**	.**034**	0.257	.303
Insulin 25 µU/mL*^d^*	0.251	.316	0.179	.478	0.052	.839	0.028	.913	0.379	.121	0.189	.453
Insulin 1000 µU/mL*^d^*	0.230	.358	0.158	.531	−0.003	.990	−0.020	.938	**0**.**474**	.**047**	0.265	.287

Correlations are Spearman rho. N = 67, unless otherwise stated. Statistically significant correlation values are shown in bold, *P* less than .05; and italics, *P* less than .1.

Abbreviations: BMI, body mass index; DHEAS, dehydroepiandrosterone sulfate; E2, estradiol; FAI, free androgen index; FEI, free estrogen index; HbA_1c_, glycated hemoglobin A_1c_; HOMA-IR, homeostatic model assessment of insulin resistance; SHGB, sex hormone–binding globulin; WHR, waist-hip ratio.

^*a*^n = 49.

^*b*^n = 19.

^*c*^n = 22.

^*d*^n = 18.

### *ARO*, *ESR1*, and *ESR2* Gene Expression in Subcutaneous Adipose Tissue Compared to Visceral Adipose Tissue

**Figure 5.**
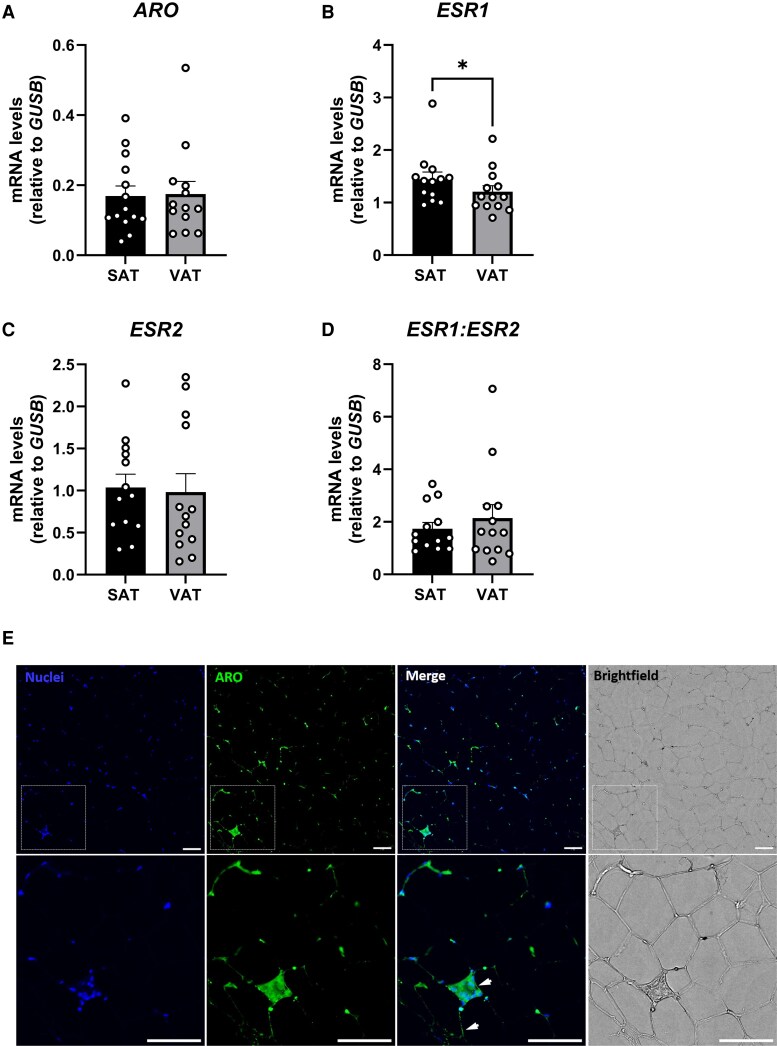
Expression of *ARO*, *ESR1*, and *ESR2* in subcutaneous adipose tissue (SAT) and visceral adipose tissue (VAT). Gene expression of A, *ARO*; B, *ESR1*; C, *ESR2*; and D, *ESR1:ESR2* in paired SAT and VAT surgical samples. Cohort 3, n = 13. E, Immunofluorescence staining of ARO protein and nuclei in SAT histological samples, 20×magnification (representative image, n = 2). Scale bars indicate 100 μm. Lower panel shows zoom of upper panel sections outlined with dashed squares. Presence of ARO indicated with white arrowheads in merged image (lower panel). *P less than .05. Data are mean ± SEM.

Since markers of adiposity were found to be the strongest predictors of *ARO* and *ESR1* gene expression in SAT, we assessed whether these genes are differentially expressed in SAT compared to VAT. *ARO*, *ESR2*, and *ESR1:ESR2* gene expression were comparable between depots (Fig. 5A, 5C, and 5D), whereas *ESR1* gene expression was lower in VAT than SAT (Fig. 5B; *P* < .05).

Additionally, immunostaining of ARO protein in SAT showed that nonadipocyte cells had the highest aromatase protein expression, but protein expression could also be detected in adipocytes, but to a lesser extent (Fig. 5E), which is in line with the findings reported by others (25, 26) and publicly available databases (27).

### Association of *ARO* SNVs With Adiposity

We analyzed the association between *ARO* SNVs and adiposity in men. *ARO* SNV rs727479 was significantly associated with WHR adjusted for BMI. Additionally, several other SNVs were linked to various adiposity-related traits, including waist and hip circumference (Table 5).

**Table 5.** Association between *ARO* single nucleotide variation and markers of body fat distribution in men

Trait	Variant ID	Location	Ref to Alt	*P*	Context	OR	95% CI
Waist-hip ratio adjusted for BMI	rs727479	Chr15:512423501242350	C/A/G/T	7.00e-11	Intron variant	0.0213524	(0.015-0.028) unit increase
Waist hip index	rs727479	Chr:15:512423501242350	C/A/G/T	5.00e-11	Intron variant	0.021577	(0.015-0.028) unit increase
Waist circumference adjusted for BMI	rs726547	Chr:15:512379701237970	G/A	1.00e-8	Intron variant	0.0424922	(0.028-0.057) unit decrease
Hip circumference adjusted for BMI	rs10459592	Chr:15:512439441243944	T/G	2.00e-12	Intron variant	0.0218048	(0.016-0.028) unit decrease
Hip circumference adjusted for BMI	rs3759811	Chri:15:512370681237068	T/A/C	5.00e-20	Intron variant	0.0280865	(0.022-0.034) unit decrease

Abbreviations: Alt, alternate; BMI, body mass index; OR, odds ratio; Ref, reference.

### Effect of Estradiol and Testosterone on Adipocyte Glucose Uptake

**Figure 6.**
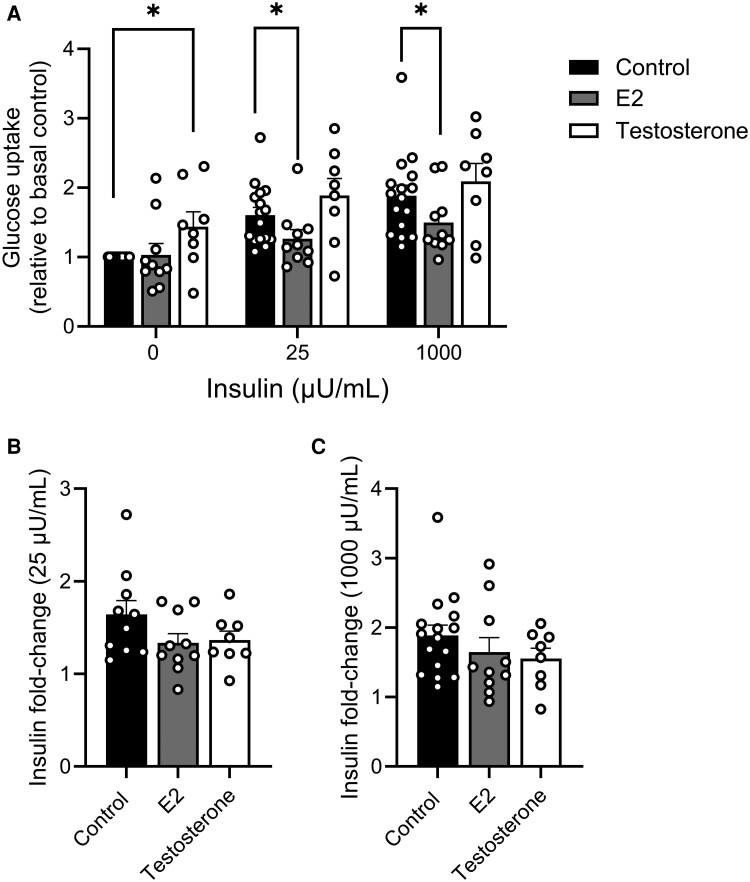
Effect of estradiol (E2) and testosterone on adipocyte glucose uptake. A, Basal and insulin-simulated glucose uptake in adipocytes isolated from subcutaneous adipose tissue from men incubated for 24 hours ex vivo with or without 0.1 nM E2 or 100 nM testosterone and the fold change by B, 25 µU/mL, and C, 1000 µU/mL insulin, respectively, was compared to the basal condition. Data are mean ± SEM, from men with no diabetes (body mass index 23.7-43.8), from cohort 1. E2, n = 10; testosterone, n = 8. The relative increase by insulin was log-transformed prior to statistical analysis. *P less than .05.

The effect of E2 and testosterone on basal and insulin-stimulated adipocyte glucose uptake was investigated. Following a 24-hour incubation of SAT from men with either 0.1 nM E2 or 100 nM testosterone, E2 reduced insulin-stimulated glucose uptake in adipocytes by 23% (Fig. 6A, 25 µU/mL and 1000 µU/mL; both *P* < .05). In contrast, testosterone increased basal glucose uptake 1.4-fold (Fig. 6A; *P* < .05). However, neither E2 nor testosterone significantly altered the insulin-stimulated fold increase compared to basal conditions (Fig. 6B and 6C, respectively).

## Discussion

In this study, we investigated the expression of ARO, ESR1, and ESR2 in SAT and VAT from men with varying degrees of obesity and T2D, as well as the effects of the sex steroids E2 and testosterone on adipocyte glucose uptake. Our findings provide novel insights into how local estrogen metabolism in adipose tissue may contribute to obesity-related metabolic dysfunction. Specifically, we demonstrate that *ARO* gene and protein levels are elevated in SAT from men with obesity, and that aromatase is associated with markers of central adiposity, as well as insulin resistance. Importantly, we observed no association between ARO expression and circulating E2 levels, suggesting a disconnect between systemic and local estrogen effects. We also show that *ESR1* and *ESR1:ESR2* ratio, but not *ESR2*, gene expression levels in SAT are decreased in men with obesity. Furthermore, we provide the first evidence that E2 directly impairs insulin-stimulated glucose uptake in SAT from men, a potential mechanistic link between aromatase activity and metabolic dysregulation in obesity.

Our findings that ARO gene and protein expression in SAT were higher in men with obesity compared to nonobese individuals, and positively correlated with obesity markers such as BMI, body fat percentage, and WHR, align with previous studies showing a positive association between BMI and increased *ARO* gene expression in SAT (28, 29). ARO converts androgens to estrogens, and approximately 80% of E2 in men is produced through the aromatization of testosterone in extragonadal tissues, including adipose tissue (30). While elevated circulating estrogen levels in men have been linked to obesity and, in particular, visceral adiposity (7), not all studies have demonstrated that obesity leads to increased E2 levels (31-33). In our study, we found no association between *ARO* gene expression and circulating E2 levels, which suggests that estrogen production in adipose tissue may not always reflect systemic levels. Our study did show a negative association between circulating testosterone and several obesity markers, including BMI, as well as lower testosterone/E2, which are consistent with findings that obesity is associated with reduced total testosterone (33, 34) and an elevated E2/testosterone ratio (35). Increased ARO activity likely contributes to this imbalance by converting more testosterone into E2. Supporting this, treatment with aromatase inhibitors or downmodulators has been shown to improve the E2/testosterone ratio in circulation (7, 9, 36), and to enhance body fat reduction when combined with weight loss compared to weight loss alone (7). High aromatase levels in adipose tissue suggest that more testosterone is converted to E2 locally, which could partly explain the negative correlation between circulating testosterone and BMI in our study, as previously suggested by others (8, 9). However, as stated earlier, this does not seem to cause a concomitant elevation of circulating E2 levels. It has previously been shown that E2 concentrations in adipose tissue can be more than double that of the concentration in serum (29), implying that local estrogen production within adipose tissue may have considerable metabolic effects independent of systemic E2 levels. More studies are needed to investigate how circulating and local tissue concentrations of these hormones are affected by aromatization in adipose tissue.

E2 exerts its metabolic effects primarily through the ESR1 and ESR2 receptors, and these effects are partly determined by the ratio of these receptors (37, 38). In our study, we observed reduced *ESR1* gene expression in SAT from men with obesity, along with a trend for lower *ESR1* gene and protein expression in SAT from men with T2D compared to those without T2D. Additionally, *ESR1* gene expression was negatively correlated with markers of obesity (eg, BMI, WHR), impaired glycemia, and insulin resistance (eg, HbA_1c_, HOMA-IR). This is consistent with a previous study showing that *ESR1* gene expression is reduced in SAT from men with obesity, but unaltered by T2D (38). Moreover, Colleluori et al (39) found that hypogonadal men with obesity had lower *ESR1* gene expression in SAT compared to nonobese men, and higher E2 serum levels in these men were associated with lower *ESR1* expression in SAT. This suggests a potential inverse relationship between ESR1 and ARO. We observed a similar pattern in our study, with *ARO* gene expression negatively associated with *ESR1* gene expression and *ESR1*:*ESR2* ratio in SAT. However, we did not find any correlation between serum levels of E2, neither total nor free, and *ESR1* or *ARO*. This discrepancy might be explained by cohort differences, as our cohort did not include men with hypogonadism or elevated E2 levels.

ESR1-specific signaling in adipocytes has been shown to promote insulin sensitivity (10-12), and reductions in ESR1 have been linked to obesity and insulin resistance (40, 41). In our study, ESR1 tended to be lower in SAT from men with T2D, which may contribute to impaired glucose metabolism and insulin sensitivity in these individuals. Although we observed no statistically significant changes in *ESR2* gene or protein expression in SAT from men, either due to obesity or T2D status, the negative correlation between *ESR2* gene expression and WHR, along with the positive correlation with testosterone levels, suggests a role of ESR2 in body fat distribution. While these correlations do not indicate significant changes in ESR2 expression, they still highlight its potential involvement in regulating visceral fat accumulation. This finding aligns with previous studies showing that ESR2 influences insulin sensitivity in adipocytes from women (42) and plays a role in body fat distribution in women (43, 44). It is also important to note that the discrepancy between *ESR1* gene and protein expression in the obesity comparisons raises important questions about posttranscriptional regulation and protein stability. This suggests that gene expression alone may not fully reflect the functional state of these receptors.

In this study, markers of adiposity, specifically WHR and body fat percentage, were the strongest predictors of *ARO*, *ESR1*, and *ESR2* gene expression in SAT. While *ARO* gene expression was also positively associated with markers of impaired glycemia and insulin resistance, adiposity markers remained the dominant predictors. Additionally, *ARO* gene SNVs in men were associated with WHR, further reinforcing the connection between adiposity and this gene.

When assessing depot-dependent differences, only *ESR1* showed lower gene expression in VAT compared to SAT, while *ARO*, *ESR2*, and *ESR1:ESR2* ratio did not show significant differences between depots. This contrasts with previous studies in women, where *ESR1* gene expression was higher in VAT than in SAT (14, 38), and *ESR2* was lower in VAT compared to SAT. Other studies have also demonstrated that *ESR1* gene expression in SAT and VAT is influenced by sex, age, and BMI (45). Additionally, in men, the *ESR1:ESR2* ratio has been shown to be lower in SAT compared to VAT and *ARO* expression reduced in VAT compared to SAT (29), but only in obese participants. This discrepancy might be explained by the fact that the men participating in our paired analyses were primarily nonobese, suggesting that obesity may differentially affect gene expression between depots. The positive correlation of *ARO* expression in SAT with BMI further supports this, although BMI-related changes in ARO expression in VAT remain to be elucidated. Future studies should include men with a wide BMI range to provide further insight into depot differences in ARO expression related to obesity.

Since *ARO* and *ESR1* gene expression in SAT were associated with markers of glycemia and insulin resistance, we explored their potential roles in regulating glucose uptake and lipid metabolism. Correlation analyses suggest that higher *ARO* expression is linked to reduced adipocyte insulin sensitivity and lipolysis capacity, potentially favoring lipid storage. These findings align with the positive associations observed between ARO expression and clinical markers of insulin resistance and obesity. However, it is important to acknowledge that previous studies have reported contrasting findings. Ohlsson et al (46) demonstrated that ARO overexpression in adipose tissue improved insulin sensitivity in male mice. This discrepancy probably reflects differences between species, as commonly seen in the regulation of metabolism. Previous human studies also illustrate this complexity, as aromatase inhibition has been associated with reduced insulin sensitivity in postmenopausal women (47) and healthy men (48), while increased aromatase activity in VAT was associated with metabolic dysfunction in premenopausal obese women (49). In the studies by Gibb et al (47, 48), aromatase inhibition was on a systemic level, and in men it was suggested that the worsening of insulin sensitivity with aromatase inhibition was likely through changes in local E2 generation and action at the level of the muscle (48). These findings suggest that the effects of adipose aromatase on metabolic regulation are dependent on species, aging, fat depot, and adiposity level.

*ESR1* and *ESR2* gene expression in SAT were positively associated with ex vivo adipocyte glucose uptake and expression of key genes involved in insulin signaling and lipolysis. Interestingly, *ESR1* and *ESR2* also correlated positively with expression of lipogenesis genes, suggesting increased lipid turnover. Activation of ESR1, in particular, has been shown to promote insulin sensitivity and stimulate glucose uptake in both in vitro and in vivo rodent models (10, 11, 50), further supporting its role in adipocyte function. The interplay between aromatase activity and estrogen receptor signaling adds complexity to adipose tissue metabolism. For example, in a previous study, we found that E2 incubation of SAT from postmenopausal but not premenopausal women reduced basal and insulin-stimulated adipocyte glucose uptake, and that this reduction was partially reversed by using an ESR2 antagonist (14). Extending these findings, we also observed that E2 decreased insulin-stimulated glucose uptake in SAT from men. However, while testosterone increased basal glucose uptake, it did not change insulin-stimulated glucose uptake. This may be due to testosterone acting through alternative pathways, such as the androgen receptor, which could counteract the effects of its conversion to estrogen. For instance, testosterone increases GLUT4-dependent glucose uptake in murine adipocytes in vitro (51) and GLUT4 expression correlates positively with androgen receptor expression in rabbit adipocytes (52). Whether exposure to testosterone can have dual and opposing effects on insulin action in SAT, via the androgen receptor and via ESRs after conversion to estrogen, remains unclear, and future studies are warranted.

This study has some limitations. Discrepancies between gene and protein expression of ARO and ESR1, as has also been reported by others (45), may be due to factors such as differences in clinical characteristics of participants in the analyses, in addition to differences in posttranslational modifications and altered protein degradation. Furthermore, studies including investigations of ARO activity and concentrations of estrogens in adipose tissue, together with circulating sex hormone levels, are necessary to get a comprehensive understanding of the relationship between these parameters and the development of obesity and insulin resistance. Additionally, due the limited number of individuals with T2D, as well as participants used for testosterone and E2 incubations of SAT, subgroup correlation analysis was restricted, and few conclusions specific to these groups can be drawn. The study was cross-sectional and inference of causality is not possible. This needs to be addressed by further studies, for example, using pharmacological or genetic manipulations.

In conclusion, aromatase gene and protein expression was elevated in subcutaneous adipose tissue in men with obesity and was associated with central adiposity, but also with insulin resistance and dysglycemia. Additionally, aromatase gene expression was increased in men with T2D, and they also had a trend to lower *ESR1* expression. The data support a link between metabolic dysregulation and altered estrogen production and action in adipose tissue. We suggest that elevated aromatase content in SAT, together with altered *ESR1/ESR2* balance, in men with obesity contributes to the development of insulin resistance and T2D. Further exploration of these pathways may provide novel pharmacologic targets for cardiometabolic disorders.

## Data Availability

Data sets generated and/or analyzed in the present study can be provided by the corresponding author on reasonable request.

## Abbreviations

ARO: aromatase
BMI: body mass index
DHEAS: dehydroepiandrosterone sulfate
E2: estradiol
ESR1: estrogen receptor α
ESR2: estrogen receptor β
FAI: free androgen index
FEI: free estrogen index
FSH: follicle-stimulating hormone
HbA_1c_: glycated hemoglobin A_1c_
HDL: high-density lipoprotein
HOMA-IR: homeostatic model assessment of insulin resistance
LDL: low-density lipoprotein
LH: luteinizing hormone
ND: no diabetes
P: plasma
PBS: phosphate-buffered saline
S: serum
SAT: subcutaneous adipose tissue
SHGB: sex hormone–binding globulin
qPCR: quantitative polymerase chain reaction
T2D: type 2 diabetes
VAT: visceral adipose tissue
WHR: waist-hip ratio
